# An Account of Two Cases of Popliteal Aneurism

**Published:** 1794

**Authors:** Thompson Forster

**Affiliations:** Surgeon on the Staff of the Army, and Surgeon to Guy's Hospital.


### MEDICAL FACTS

AND

### OBSERVATIONS.

I. *An Account of two Cases of Popliteal Aneurism; communicated in a Letter to* Samuel Foart Simmons, *M. D. F. R. S. by Mr.* Thompson Forster, *Surgeon on the Staff of the Army, and Surgeon to Guy's Hospital.*

*To Dr.* SIMMONS.

DEAR SIR,

THE mode of operation for the popliteal aneurism, adopted by that truly-ingenious physiologist, the late Mr. John Hunter, may perhaps be considered as one of the most valuable improvements of modern surgery. It appears evidently to have been the refult of a judicious chain of reafoning, founded on a thorough knowledge of the vafcular fyftem, and of the powers refiding in the abforbents; but the full extent of its merits or defects, and confequently the grounds on which it may be fufceptible of farther improvement, can be afcertained only by the accumulated obfervations of different practitioners, accurately and candidly related. Hence it is that having lately had two cafes under my care, in which I have performed the operation in queftion, I confider it as a fort of duty to communicate them to the Public: I therefore take the liberty of tranfmitting to you the following account of them, to be inferted, if you deem it fufficiently interefting, in your valuable collection of Medical Facts and Obfervations.

Believe me, Dear Sir,

Yours, &c.

*October* 22, 1793.

Thompson Forster.

CASE

### CASE I.

Joſeph Keeping, aged thirty-five years, a ſtrong, healthy, hard-working man, by trade a carpenter, was admitted as my patient into Guy's Hoſpital, on the 17th of Auguſt, 1791, for the cure of an aneuriſm of the popliteal artery.

This complaint had begun about a year before, with a ſudden pain in the calf of his leg, followed by a ſlight univerſal ſwelling of the whole limb, but not attended with ſufficient inconvenience to hinder him from working daily at his buſineſs.

At the end of a fortnight he perceived a ſmall tumour more immediately in the ham; and in the courſe of about ſix weeks from its firſt appearance, it increaſed to the ſize of half a golden pippin, forming a protruſion which appeared, in ſome meaſure, diſtinct from the general œdematous enlargement of the limb.

To this tumour he applied embrocations and oils recommended by different perſons of his acquaintance, for two months more, at the expiration of which time, he was ſo far from being better, that it was not without great pain he could attend his buſineſs, the limb being more univerſally enlarged, and the difficulty of moving it ſo much increaſed, that he now found himſelf obliged to deſiſt intirely from all manner of work. In this ſtate he remained ſome time longer, with the hope that reſt, which gave him eaſe, would in time cure his complaint. He ſtill continued, however, to rub the limb with oils of ſome kind or other, till at length his hand, by frequently going over the ſmall tumour in the ham, became ſenſible of a pretty ſtrong pulſation in it. Being much alarmed at this, he came into Guy's Hoſpital in December, 1790, where reſt, an horizontal poſition, and the continued uſe of a bandage from the foot to the middle of the thigh, conſiderably diminiſhed the general enlargement of the limb, but the protruding pulſating tumour remained in the ſame ſtate as before.

As he was very deſirous of returning to his buſineſs, and no immediate danger of his life was then apprehended, he was permitted, towards the end of January, 1791, to go out of the hoſpital, as he worked near it, but was deſired to come again whenever the complaint ſhould

ſhould give him any inconvenience, and was directed to favor the limb as much as he could, and to keep it conſtantly rolled from the foot upwards. We heard nothing of him till Auguſt 17, 1791, when he appeared to be in a very alarming ſtate. His foot, leg, and ham, were now very much enlarged, and the tumour, before mentioned, was increaſed and tenſe; the ſkin was very thin, exhibiting that appearance which an abſceſs acquires when in a ſtate of perfect ſuppuration; and the pulſation of the tumour was viſible, even at ſome diſtance from him; his pulſe was very quick and full; he was extremely irritable and weak; he had a dry ſkin, with conſiderable thirſt; and anxiety was ſtrongly marked in his countenance.

I immediately ordered ten ounces of blood to be taken from his arm, and a purgative medicine to be given in the morning, as he had not had any evacuation for four days.

On the morning of the 22d, in conſultation with my colleagues, Meſſrs. Lucas and Cooper, it was judged adviſable, in order to preſerve his life, that the femoral artery ſhould be ſecured, and to truſt to the abſorbing powers to leſſen the tumour and the enlarged limb.

I placed a tourniquet on the limb as high as possible, in order to have room enough; but the tourniquet was intirely loose, and of course made no pressure on the artery. It was so placed, however, as to be capable of instantly stopping the circulation if required. I then made an incision in the course of the under edge of the sartorius muscle, about three inches in length, and, by raising up the lower edge of that muscle, I came at the artery, about two inches before it perforates the triceps femoris; after carefully separating it from the vein and nerve, I passed a broad ligature by means of a common eyed-probe under it, placing a dossil of lint on the artery immediately over the ligature, upon which lint I laid a cylindrical piece of wood, about a third of an inch in diameter, and three quarters of an inch long, so that on tying the ligature, the artery, lint, and stick became included in such a manner as to make the artery spread itself more than half round the stick thus cushioned with the dossil of lint.

I conceive all this precaution to be absolutely necessary to guard against the circumstance of the coats of the artery being cut through by the ligature in the first instance; and, in the second, to prevent the impetus of the blood from throwing throwing off the ligature at the end of ſome days, when the ulcerative proceſs may have weakened the coats of the artery at the compreſſed part: and if we allow a poſſibility of this happening before the depoſit of coagulable lymph ſhall have become ſufficiently vaſcular to render it capable of reſiſting the force of the column of blood oppoſed to it, every precaution ſhould be taken to prevent an hæmorrhage from ſo large a veſſel, which moſt likely would prove fatal before aſſiſtance could be procured; theſe were my reaſons for interpoſing the lint and ſtick between the ligature and the artery. I then drew the ligature tight enough to ſtop all pulſation in the tumour below; I left the ends of the ligature out of the wound, which I partially cloſed; the dreſſings were ſuperficial, and I applied an eaſy bandage.

The firſt conſequence reſulting from this ligature, was an immediate ſtop to the pulſation in the tumour, and an evidently-increaſed fulneſs in the pulſe at the wriſt. The patient was kept perfectly quiet, and allowed only diluting drinks; at eight in the evening, (about ſeven hours after the operation) his pulſe being quick and full, ſix ounces of blood were taken from his arm, and he took a grain of opium.

Aug. 23d. He had paſſed a reſtleſs night, with thirſt, and pain in his thigh. The temperature of the limb was this day ſeveral degrees lower than that of the other limb; but as I ſubjoin a table of temperature, I ſhall omit it in the narrative; his pulſe, however, was more moderate; he had ſome good ſleep during the day, and his pain became leſs, but his pulſe was quicker towards the evening; the opiate was repeated.

24th. This morning he ſcarcely complained of any pain in the limb. As he was coſtive, I ordered him ſome caſtor oil, by means of which ſtools were procured.

The next day, (the 25th) he was very calm, and without any pain in the limb, having ſlept well the preceding night; ſtill, however, his pulſe kept at from 102 to 110. I gave him a ſaline julep and the opiate as before.

Except a quickneſs of his pulſe, which came on every evening, coſtiveneſs, which was occaſionally relieved, and a certain irritability about him, nothing occurred till the 27th, when an uneaſineſs and fætor in the limb obliged me to dreſs the wound. There was then a very great diſcharge of good matter; I ordered him the bark, and a more nouriſhing diet. From this this time he loſt that quickneſs of his pulſe, and that irritability before mentioned; his limb, below the ligature, about this time became relaxed, particularly the tumour in the ham; the ſize of the whole limb was perceptibly leſs, and his appetite returned; but the diſcharge remaining very conſiderable, he continued to take the bark and opium, and care was taken to obviate coſtiveneſs.

On the eighth of September, that is, on the ſeventeenth day from the operation, the ligature, ſtick, and lint came all away together, without the leaſt pain or force; ſo that a total ſolution of the continuity of the artery muſt have taken place. The limb, at this time, meaſured two inches leſs in circumference than it did before the operation. Embrocations and a moderately-tight bandage of flannel were daily uſed from the toe to the wound.

From the time the ligature came away, the diſcharge gradually leſſened, and in the courſe of the month the wound was nearly healed; but it then putting on an ulcerating appearance, and ſoon after ſpreading cutaneouſly over a conſiderable portion of the thigh, I conceived that country air, and moderate exerciſe, might be highly beneficial to the patient. I accordingly accordingly procured him a lodging at Lambeth, where I occafionally attended him, and, in a months refidence there, the whole ulcerated furface was intirely healed, and by gradual and moderate exertions he became able to work at his bufinefs as well as ever. I faw him about a week ago, and examined the limb, which was fcarcely perceptibly larger than the other. The tumour in the ham, though much diminifhed, remains in a flaccid ftate, without the leaft pulfation or pain; and he tells me he can ufe it in every refpect as well as ever he could in his life.

The comparative heat of the found and of the difeafed limb was carefully afcertained by means of a thermometer every evening*, for fome days after the operation, and found to be as follows:

* On the evening of the day of the operation, the difeafed limb was found to be fome degrees colder than the other; but as the exact degree of heat was omitted to be noted at the time, it could not be inferted in the table. The bulb of the thermometer (Fahrenheit's) employed in thefe obfervations, was flat. It was applied each day on the ham and foot, and was continued for fome minutes on thofe parts before the degree of heat could be fatisfactorily obtained.

Days

| Days after the Operation | Temperature of the found Limb. At the Ham. | Temperature of the found Limb. At the Foot. | Temperature of the difeafed Limb. At the Ham. | Temperature of the difeafed Limb. At the Foot. |
|---|---|---|---|---|
| 1ft. | 96° | 96° | 95° | 92° |
| 2d. | 94° | 93° | 97° | $94\frac{1}{2}$° |
| 3d. | 94° | 94° | 99° | 96° |
| 4th. | 95° | 94° | 99° | 95° |
| 5th. | 94° | 93° | 98° | 95° |
| 6th. | $95\frac{1}{4}$° | 94° | 98° | 96° |

The temperature was thus taken for three weeks, and it was obferved, that the difeafed limb became gradually lefs warm, compared with the other; and that at the end of that period both limbs became equal in heat.

### CASE II.

Nicholas Tatterfhall, aged 37 years, was admitted into Guy's Hofpital, under my care, on the 5th of June, 1793, for the cure of a large pulfating tumour, which occupied the whole of the hollow of the left ham, and appeared anteriorly in fuch a manner, as almoft to furround the lower part of the thigh; the leg and foot were alfo much enlarged and hard; the knee retained its motion, though every exertion of it was attended with pain in the tumour. The patient himfelf afcribed the origin of his complaint plaint to a ſevere blow from the helm in a ſtorm at ſea, which had diſabled him for a fortnight; and after that he had frequently, he ſaid, felt pain on any quick motion of the limb. It was about ſix weeks after the blow that he firſt diſcovered a ſmall throbbing ſwelling in the ham, preciſely at the place where he had ſo frequently felt the acute pain before mentioned; and this tumour had kept gradually increaſing in ſize and hardneſs till the time I ſaw him, which included a ſpace of fourteen months.

On carefully examining the tumour, and collecting the preceding account of it from the patient, I had no difficulty in pronouncing it to be an aneuriſm of the popliteal artery.

The patient being a very athletic ſtrong man, I ordered fourteen ounces of blood to be taken from his arm; an aperient medicine to be given him twice a week; and the warm bath to be had recourſe to every other day. I moreover put him upon an abſtemious diet. By purſuing this treatment for three weeks, he was conſiderably lowered and relaxed, and conſequently leſs liable to any high degree of inflammatory action; the tumour in the ham, however, and the general enlargement of the limb, remained much in the ſame ſtate as at his admiſſion.

Thinking him now in a favourable ſtate for an operation, I propoſed it to my colleagues in the hoſpital, Meſſrs. Lucas and Cooper, for whoſe opinions I entertain the higheſt reſpect. They intirely agreed with me in the neceſſity of an operation; and as I had ſo recently ſucceeded in tying the artery in the middle of the thigh, I propoſed that mode, to which they likewiſe aſſented, and they thought with me, that this was a very favourable caſe for ſuch an operation. I accordingly performed it on the 24th of June, preciſely in the ſame manner as in the preceding caſe; with this difference, however, in the reſult, that two muſcular branches of the artery were divided, and ſecured with the ligature and tenaculum before the main trunk was laid bare; I then opened the faſcia enveloping the femoral artery, and having paſſed a double ligature in an eyed probe with eaſe under it, applied the lint and ſtick as before; and for the ſame reaſons, which need not be here repeated, one ligature was drawn tight upon the artery, ſtick, and lint, and the other was placed about half an inch higher upon the artery than the other, and left looſe in order to be tied in caſe of hæmorrhage.

The ſymptoms of inflammation were much milder than in the former caſe, probably owing to the the precautions which I had taken to lower the patient during three weeks before the operation; for where the urgency of a caſe does not make an operation immediately neceſſary for the preſervation of the patient's life, I think it adviſable, previouſly to this, as well as to moſt other capital operations, to reduce the patient to that ſtate in which he is the leaſt liable to ſuffer from ſubſequent inflammation.

On the ſixth day after the operation, I found it neceſſary to dreſs the wound; the diſcharge was great, (but by no means equal in quantity to that in the former caſe) much leſs fætid, and the edges of the wound had a diſpoſition to heal; but the ligature, of courſe, prevented them.

As a knowledge of the comparative temperature of the limb operated upon, with that of the ſound one, the ſtate of the patient's pulſe, and of the atmoſpheric heat, were objects I conceived to be both uſeful and curious, I was anxious to have them accurately attended to. This taſk was readily undertaken by Mr. William Hall, of Glouceſter, who was then a pupil at the hoſpital, and on whoſe accuracy I knew I could rely. The annexed table, which contains the reſult of his obſervations, may therefore be be depended on. As the atmofpheric heat is perpetually varying, I thought we fhould arrive at a greater degree of exactnefs if we prevented the accefs of the common air to the bulb of the thermometer during the time of its application to the limb, by covering both limb and thermometer with flannel. This was carefully attended to every day, till both limbs fhewed the fame temperature, which they did in about a month.

After the firft opening of the wound, it became neceffary to drefs it every day. On the fifth of July, the ligatures from the mufcular arteries came away; and on the fixteenth, the ligature, ftick, and lint came all away together, as in the foregoing cafe. I permitted the fpare ligature to remain till the twenty-third, when conceiving that it occafioned irritation, and of courfe excited difcharge, I withdrew it; from this time the difcharge gradually diminifhed, and the wound was healed on the 28th of Auguft. The fwelling in the ham at this time was fcarcely vifible; the leg and foot were become of their natural fize, and the patient ufed that limb as freely as the other. He was difmiffed the hofpital, and entered directly on board a fhip of war.

TABLE

### TABLE.

| Day of the Month. | Right Ham. | Left Ham. | Right Foot. | Left Foot. | Tempe-rature of the At-mosfphere. | Time of Day when the Obfervations were made. | Pulfe at the wrift. |
|---|---|---|---|---|---|---|---|
| | Temp. | Temp. | Temp. | Temp. | | | |
| June 24 | 96 ° | 95 ° | 79 ° | 78 ° | | 3 o'clock, P. M. | 80 |
| | 96 | 95 | 90 | 91 | | 10 ——— P. M. | 140 |
| 25 | 99 | 98 | 93 | 95 | | 9½ ——— — — | 94 |
| 26 | 102 | 96 | 93 | 82 | | 9½ ——— — — | 100 |
| 27 | 98 | 94 | 88 | 81 | 66 ° | 10 ——— — — | |
| 28 | 101 | 97 | 94 | 91 | 70 | 9½ ——— — — | 105 |
| 29 | 99 | 96 | 93 | 90 | 72 | 9¼ ——— — — | 92 |
| 30 | 99 | 95 | 91 | 81 | 67 | 10 ——— — — | 86 |
| July 1 | 98 | 95 | 94 | 84½ | 67 | 9¼ ——— — — | 72 |
| 2 | 99 | 97 | 93 | 88 | 69 | 9½ ——— — — | 74 |
| 3 | 98 | 97 | 95 | 90½ | 70 | 9½ ——— — — | 82 |
| 4 | 98½ | 97 | 95 | 93 | 69 | 10 ——— — — | 70 |
| 5 | 97 | 93 | 92 | 92 | 74 | 9½ ——— — — | 88 |
| 6 | 101 | 99 | 97½ | 99 | 79 | 9½ ——— — — | 88 |
| 7 | 100 | 98 | 97 | 97 | 81½ | 10 ——— — — | 84 |
| 8 | 99 | 96½ | 96 | 95 | 71 | 10 ——— — — | 74 |
| 9 | 98 | 97 | 94 | 93 | 70 | 10 ——— — — | 78 |
| 11 | 100 | 97 | 97¾ | 96 | 75 | 10 ——— — — | 80 |
| 12 | 99 | 98 | 97 | 96 | 75 | 10 ——— — — | 84 |
| 13 | 99 | 98 | 95¾ | 95 | 70 | 10 ——— — — | 86 |
| 15 | 101 | 98 | 96½ | 94 | 74 | 10 ——— — — | 86 |
| 16 | Ligatures came a-way. | | | | | | |
| 17 | 100 | 97 | 98 | 96 | 75¾ | 10 ——— — — | 86 |
| 18 | 99 | 97 | 95 | 94 | 69 | 10 ——— — — | 74 |
| 19 | 97 | 96 | 90 | 82 | 68 | 10 ——— — — | 84 |
| 21 | 97 | 95 | 88 | 80 | 66 | 10 ——— — — | 72 |
| 24 | 94 | 92 | 93 | 89 | 75 | 8 ——— — — | 90 |
| 25 | 97¼ | 97 | 94 | 93 | 73 | 8 ——— — — | 80 |
| 27 | 98 | 97 | 93 | 85 | 66 | 9½ ——— — — | 86 |
| 29 | 98 | 98 | 84 | 80 | 66½ | 8 ——— — — | 90 |
| 30 | 98 | 98 | 94 | 94 | 67 | 9½ ——— — — | 86 |

II. *An*

